# Promising Bialkali Bismuthides Cs(Na, K)_2_Bi for High-Performance Nanoscale Electromechanical Devices: Prediction of Mechanical and Anisotropic Elastic Properties under Hydrostatic Tension and Compression and Tunable Auxetic Properties

**DOI:** 10.3390/nano11102739

**Published:** 2021-10-16

**Authors:** Shahram Yalameha, Zahra Nourbakhsh, Ali Ramazani, Daryoosh Vashaee

**Affiliations:** 1 Faculty of Physics, University of Isfahan, Isfahan 81746-73441, Iran; yalameha93@gmail.com (S.Y.); z.nourbakhsh@sci.ui.ac.ir (Z.N.); 2 Department of Mechanical Engineering, Massachusetts Institute of Technology, 77 Massachusetts Ave., Cambridge, MA 02139, USA; ramazani@mit.edu; 3 Department of Electrical and Computer Engineering, North Carolina State University, Raleigh, NC 27606, USA; 4 Department of Materials Science and Engineering, North Carolina State University, Raleigh, NC 27606, USA

**Keywords:** mechanical properties, elastic anisotropy, negative Poisson’s ratio, auxetic material

## Abstract

Using first-principles calculations, we predict highly stable cubic bialkali bismuthides Cs(Na, K)_2_Bi with several technologically important mechanical and anisotropic elastic properties. We investigate the mechanical and anisotropic elastic properties under hydrostatic tension and compression. At zero pressure, CsK_2_Bi is characterized by elastic anisotropy with maximum and minimum stiffness along the directions of [111] and [100], respectively. Unlike CsK_2_Bi, CsNa_2_Bi exhibits almost isotropic elastic behavior at zero pressure. We found that hydrostatic tension and compression change the isotropic and anisotropic mechanical responses of these compounds. Moreover, the auxetic nature of the CsK_2_Bi compound is tunable under pressure. This compound transforms into a material with a positive Poisson’s ratio under hydrostatic compression, while it holds a large negative Poisson’s ratio of about −0.45 along the [111] direction under hydrostatic tension. An auxetic nature is not observed in CsNa_2_Bi, and Poisson’s ratio shows completely isotropic behavior under hydrostatic compression. A directional elastic wave velocity analysis shows that hydrostatic pressure effectively changes the propagation pattern of the elastic waves of both compounds and switches the directions of propagation. Cohesive energy, phonon dispersion, and Born–Huang conditions show that these compounds are thermodynamically, mechanically, and dynamically stable, confirming the practical feasibility of their synthesis. The identified mechanisms for controlling the auxetic and anisotropic elastic behavior of these compounds offer a vital feature for designing and developing high-performance nanoscale electromechanical devices.

## 1. Introduction

The alkali, bialkali bismuthides, and bialkali antimonides are highly quantum-efficient semiconductors, attracting the attention of research communities for their applications in photodetectors, photo-emissive, and sensing technologies [1,2]. Characteristics such as photon absorption and practical work function make bialkali antimonides suitable candidates for electron emission devices [3,4,5]. The topological phases of these compounds are also studied. The cubic bialkali bismuthide of KNa_2_Bi can be driven into a topological insulator or a three-dimensional (3D) Dirac semimetal under uniaxial compression or tensile strain, respectively [6]. The cubic bialkali antimonide KNa_2_Sb can also be turned into a topological insulator under hydrostatic pressure [7]. In addition, among Bi-based alkali metal compounds, A_3_Bi (A = Na, K, Rb) belongs to a particular class of topological electronic states, 3D Dirac semimetals [8]. Experimentally, much attention has been paid to alkali antimonides to explore the critical electrical and optical properties for technological applications [9,10,11,12]. On the theoretical side, most bialkali antimonide compounds have been widely studied. For instance, Kalarasse et al. [13] investigated the structural, elastic, electronic, and optical properties of cubic bialkali antimonides, Na_2_KSb, Na_2_RbSb, Na_2_CsSb, K_2_RbSb, K_2_CsSb, and Rb_2_CsSb, using first-principle methods. Amador [14] investigated the electronic structure and optical properties of Na_2_KSb and NaK_2_Sb from the first-principles many-body theory. Vineet Kumar et al. [15] have studied the thermoelectric properties of the bialkali antimonide Na_2_KSb using the full-potential-linearized augmented plane wave. A recent study examined the nontrivial topological properties of CsNa_2_Bi and CsK_2_Bi compounds [16]. However, a comprehensive study of the stability and mechanical properties of cubic bialkali bismuthide CsNa_2_Bi and CsK_2_Bi compounds under equilibrium and hydrostatic pressure is still missing.

Most of the theoretically suggested materials are stable; however, their synthesis was not possible in some cases. One of the main reasons for the contradictory theoretical predictions is that not all the stability criteria were respected in the calculations. Generally, the essential stability criteria for a given structure can be divided into three categories: (1) A criterion arises from the total energies that must meet the conditions E_T_ (compound) < E_T_ (all elements); the difference between the two energies is called the *cohesive* energy (as a necessary condition), which must be negative; (2) the mechanical stability (as a necessary condition); a necessary condition for the thermodynamic stability of a crystal system is that the crystals must be mechanically stable against arbitrary (but small) homogeneous deformations [17]; (3) the dynamical stability (as a sufficient condition); this condition is satisfied by the phonon dispersion. The presence of imaginary frequencies in phonon dispersion leads to a violation of this criterion.

Elastic constants provide essential information concerning the strength of materials and often act as stability criteria or order parameters in investigating the problem of structural transformations [18,19]. Physical properties, such as sound velocity, hardness, Debye temperature, and the melting point, are related to the elastic constants [20,21,22]. In addition, phenomena such as a negative Poisson’s ratio (NPR), negative linear compressibility (NLC), and anisotropic mechanical response are characterized by these constants. These properties are an essential requirement for fundamental research and experimental investigations [23,24]. The anisotropic mechanical response and NPR in auxetic materials are of great interest because of the generally enhanced mechanical properties. The materials with NPR typically possess enhanced toughness, shear resistance, and efficient sound or vibration absorption, which enable various applications, such as personnel protection [25], automotive industries [26], biomedicine [27], aerospace and defense [28], and many commercial applications [29,30]. The elastic anisotropy of materials is also an important characteristic that affects other material properties, such as phase transformations [31], indentation resistance [32], plastic deformation [33], and crack propagation [34]. Therefore, the analysis of elastic anisotropy is an essential characterization of material properties.

The present report introduces so-far hypothetical cubic bialkali bismuthides Cs(Na, K)_2_Bi and investigates all the stability criteria as well as the mechanical and anisotropic elastic properties under hydrostatic tension and compression. First, we study the structural properties and stability conditions of these compounds, including the formation energy and mechanical and dynamical stability. Then, the effect of hydrostatic pressures on the mechanical behaviors, such as the anisotropic elastic property, NPR, and elastic wave velocities, is investigated. Furthermore, several polycrystalline modules involving the bulk modulus, Young’s modulus, shear modulus, Pugh ratio, and brittle/ductile characteristics will be presented.

## 2. Computational Details

Our calculations are carried out in the framework of DFT by using the WIEN2k package (v19.1, Vienna, Austria) [35]. The generalized gradient approximation (GGA) of Perdew–Burke Ernzerhof (PBE) formalism is adopted for the exchange–correlation potential [36]. The bulk BZ of these compounds is calculated using a 12 × 12 × 12 ***k***-point mesh. Furthermore, the wave function inside the muffin-tin sphere is extended in terms of spherical harmonics up to *l_max_* = 10 and the plane wave cut off R_MT_/K*_max_* = 9.5. The energy convergence criterion is set to 10^−5^ Ry, and the charge convergence is less than a 10^−3^ electronic charge in these materials. The phonon dispersion of the Cs(Na, K)_2_Bi material is computed using the all-electron FHI-aims code (v200112, Volker Blum, Berlin, Germany) [37] with the Phonopy package (v2.9.0, Kyoto, Japan) [38] within the GGA approach. For elastic constants calculations, we used the IRelast code [39]. In addition, the ElaTools code [40] was performed for the analysis of elastic constants and visualization of mechanical properties.

## 3. Results and Discussion

### 3.1. Structural Properties and Stability Conditions

**Figure 1:**
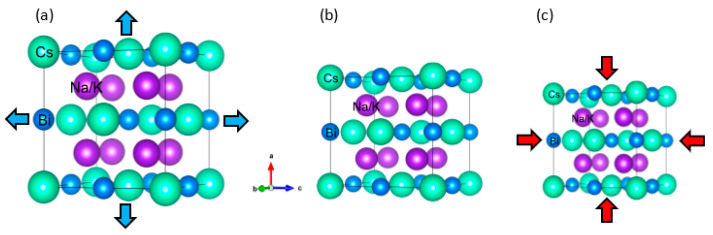
The crystal structures of Cs(Na, K)_2_Bi compounds (**a**) under hydrostatic tension, (**b**) equilibrium states, and (**c**) hydrostatic compression. The Cs atoms are sited at 4a (0, 0, 0) Wyckoff position, Na/K atoms are sited at 8c (0.25, 0.25, 0.25) and (0.25, 0.25, 0.25) Wyckoff positions, and Bi atoms are sited at 4b (0.5, 0.0, 0.0) leading to a primitive cell involving four formula units, namely two K/Na atoms and two Bi and Cs atoms.

Bialkali bismuthide Cs(Na, K)_2_Bi has a cubic crystal structure with space group Fm-3m (No. 225) (similar to full Heusler compounds [41,42]), as shown in Figure 1. In these structures, the Cs atoms are sited at the 4a (0, 0, 0) Wyckoff position, Na/K atoms are sited at the 8c (0.25, 0.25, 0.25) and (0.25, 0.25, 0.25) Wyckoff positions, and Bi atoms are sited at 4b (0.5, 0, 0) leading to a primitive cell involving four formula units, namely two K/Na atoms and two Bi and Cs atoms. The optimized values of the primitive lattice constants (*a*_0_) of these compounds are 5.86 Å (CsNa_2_Bi) and 6.32 Å (CsK_2_Bi). In the following, we will investigate all the essential stability criteria, namely, thermodynamic, mechanical, and dynamic stability of these compounds.

The cohesive energy ($E_{C}$) is calculated to determine the thermodynamic stability of the structures. The $E_{C}$, which is the necessary energy to separate the solids in atoms at stable states, was calculated using the following equation [43,44],

(1) $$E_{C}=\frac{E_{Bulk}^{Tot}-N_{Cs}E_{Cs}^{Tot}-N_{Bi}E_{Bi}^{Tot}-N_{Na/K}E_{Na/K}^{Tot}}{N_{Cs}+N_{Bi}+N_{Na/K}},$$

where $E_{Bulk}^{Tot}$ is the total energy of the bulk, and $E_{Cs}^{Tot}$, $E_{Bi}^{Tot}$, and $E_{Na/K}^{Tot}$ are the total energy of each element. Furthermore, *N*_Cs_, *N*_Bi,_ and *N*_Na/K_ are the number of atoms of each element in the unit cell.

The calculated cohesive energies for CsNa_2_Bi and CsK_2_Bi compounds are −1.70 and −1.62 eV/atom, respectively. According to the negative values of cohesive energy [44,45,46], these structures are thermodynamically stable. In addition to the cohesive energy, the enthalpy of formation (∆*_F_H*) (or formation energy (E*_f_*)) of these compounds has been calculated. The ∆*_F_H* can be defined as the difference in total energy of the compound and the energies of its constituent elements in their stable states [47]:

(2) $$\Delta_{F}H=E_{Cs{\left(Na,\;K\right)}_{2}Bi}^{tot}-E_{Cs}^{Bulk}-2E_{Na/K}^{Bulk}-E_{Bi}^{Bulk},$$

where $E_{\mathrm{Cs}{\left(\mathrm{Na},\;K\right)}_{2}Bi}^{tot}$ is the total energy per formula unit of Cs(Na, K)_2_Bi, and $E_{Cs}^{Bulk}$, $E_{Na/K}^{Bulk}$, and $E_{Bi}^{Bulk}$ are the total energies per atom of pure elements in their stable structures. If we ignore the influence of pressure on the condensed phases and calculate the energies at 0 K without any entropic contributions, the formation energy can be taken as ∆*_F_H* [47]. The calculated enthalpy formation of CsNa_2_Bi and CsK_2_Bi compounds is −44.73 kJ/mol and −26.28 kJ/mol, respectively. The results of the E_C_ and ∆*_F_H* show that CsNa_2_Bi is more stable than CsK_2_Bi. On the other hand, it was predicted (in Materials Project (MP) with mp-1096426 ID [48]) that the compound CsNa_2_Bi can be decomposed into Cs_3_Bi (cubic phase) and Na_3_Bi (hexagonal phase):

(3) $${3\mathrm{CsNa}}_{2}\mathrm{Bi}\;\;\overset{\Delta_{F}H=\;\;-17.47\;\;\mathrm{kJ}/\mathrm{mol}}{\to }\;\;{\mathrm{Cs}}_{3}\mathrm{Bi}\;\;\;+\;\;\;2{\mathrm{Na}}_{3}\mathrm{Bi}\;.$$

Based on this balanced chemical equation, the sum of product enthalpy of the formations (∆*_F_H* _products_) and reactions (∆*_F_H* _reactions_) is −231.81 kJ/mol and −214.34 kJ/mol, respectively. Therefore, the reaction enthalpy of the formation (∆*_F_H* _reaction_ = ∆*_F_H* _products_ − ∆*_F_H* _reactions_) is −17.47 kJ/mol (exothermic reaction), indicating that this compound can be decomposed into Cs_3_Bi and Na_3_Bi compounds. For the CsK_2_Bi compound, such a balanced equation is also examined:

(4) $${3\mathrm{CsK}}_{2}\mathrm{Bi}\;\;\overset{\Delta_{F}H=\;\;+2.05\;\;\mathrm{kJ}/\mathrm{mol}}{\to }\;\;{\mathrm{Cs}}_{3}\mathrm{Bi}\;\;\;+\;\;\;2K_{3}\mathrm{Bi}\;.$$

In this balanced chemical equation, the sum of ∆*_F_H* _products_ and ∆*_F_H* _reactions_ is −469.45 kJ/mol and −471.50 kJ/mol, respectively. Although the difference in enthalpy energy between products and reactants is small, it is an exothermic reaction (∆*_F_H* _reaction_ ≈ 2 kJ/mol). It should be noted that these results were calculated at the standard temperature and pressure (STP) conditions. Thus, though the energies suggest that these materials could be found at normal conditions, other stabilities may still be required to synthesize the material.

The elastic tensor was calculated to evaluate the mechanical stability by evaluating the elastic constants, which are listed in Table 1. As listed in Table 1, the elastic constants of CsK_2_Bi are in good agreement with the elastic constants in MP. In general, the Born–Huang criterion is used to illustrate the mechanical stability of the crystal structure [49]. In the case of cubic crystals, the Born–Huang conditions of stability is a simple form:

(5) $$C_{11}-C_{12}>0\;;C_{11}+2C_{12}>0\;;C_{44}>0\;.$$

The mechanical stability of the CsNa_2_Bi and CsK_2_Bi compounds show that the elastic constants of the compounds satisfy the Born–Huang criterion, i.e., they are mechanically stable.

**Figure 2:**
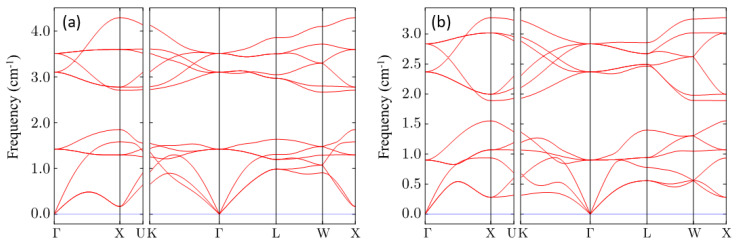
Phonon dispersion curves of the (**a**) CsNa_2_Bi and (**b**) CsK_2_Bi compounds along with high symmetry points in the Brillouin zone. It can be clearly seen that the phonon dispersion exhibits no imaginary frequency (soft phonon modes), confirming the dynamic stability of these compounds.

As a determination of the last stability condition, the dynamic response of these compounds is investigated by phonon calculation. The calculated phonon dispersions of CsNa_2_Bi and CsK_2_Bi along the high symmetry points in the Brillouin zone are shown in Figure 2a,b, respectively. No imaginary phonon frequency is found for these compounds. An imaginary phonon frequency, if it existed, would indicate that the structure is dynamically unstable (or has a phase transition) and vice versa [50]. Therefore, it is concluded that Cs(Na, K)_2_Bi materials are thermodynamically, mechanically, and dynamically stable. This proves that both compounds have a high degree of stability.

### 3.2. Basic Mechanical Properties

At the beginning of this section, we discuss the elastic constants (*C_ij_*) under hydrostatic pressures and define their relationship with the macroscopically measurable quantities that give us information about the elastic and mechanical properties of the system. In the present work, the hydrostatic pressures (i.e., hydrostatic tension and compression) are investigated according to the volume ratio V/V_0_, which is between small values of V/V_0_ = 1 ± 0.03. The corresponding hydrostatic pressures of these volume ratios for each of these compounds are presented in Table 1. The Young’s modulus (E), bulk modulus (B), shear modulus (G), and Poisson’s ratio (ν) are known as the fundamental elastic properties and are macroscopically measurable quantities that give a measure of the elasticity of the material. Voigt–Reuss–Hill (VRH) approximation [44,51,52] was utilized to calculate the four moduli (E, B, G, and ν). Table 1 shows the calculated elastic constants under pressures with V/V_0_ = 1.03 (hydrostatic tension), V/V_0_ = 1.0 (equilibrium state/zero pressure), and V/V_0_ = 0.97 (hydrostatic compression) volume ratios. It is well known that C_11_ indicates the [100] directional linear compression resistance [53], and C_44_ represents the magnitude of the [001] directional resistance on the (100) plane under the monoclinic shear stress [53,54]. This table shows that between two compounds, the C_11_ values of CsNa_2_Bi are larger than C_12_ and C_44_ in all three pressure cases, indicating that it is difficult to compress CsNa_2_Bi along the [100] direction. However, under V/V_0_ = 1.03, the C_44_ value of CsK_2_Bi is larger than C_12_ and C_11_, which indicates that this material shows higher [001] directional resistance on the (100) plane under shear deformation.

Generally, the bulk modulus (*B*) shows the compressibility of solids under hydrostatic pressure [53]. So, a larger *B* value indicates that the material is more difficult to be compressed. It can also be used as a measure of the average bond strength of atoms for given crystals. From Table 1, the CsNa_2_Bi and CsK_2_Bi have the largest and smallest bulk moduli (under V/V_0_ = 1.0 and 1.0 ± 0.03), respectively, indicating that CsNa_2_Bi and CsK_2_Bi are the most incompressible and the most compressible, respectively. Therefore, the bond strength of CsK_2_Bi should be the weakest, while that of CsNa_2_Bi should be the strongest. The shear modulus (G) is an important characteristic for resisting deformation under shear stress, and a larger G corresponds to a higher shear resistance [54]. On the other hand, G is also related to hardness, and a large shear modulus corresponds to high hardness. The CsNa_2_Bi and CsK_2_Bi compounds have the largest and smallest shear moduli, respectively, indicating that CsNa_2_Bi and CsK_2_Bi have the highest hardness and they are the highest shear resistance under shear stress, respectively. Furthermore, Young’s modulus (E) defined as the ratio of the stress to strain, is used to measure the stiffness of the solid, and when the value of E is large, the material is stiff. In this case, Young’s modulus of CsNa_2_Bi is the largest, which indicates that it has the highest stiffness. Poisson’s ratio (*ν*) and Pugh’s ratio (B/G) can be used to describe the ductility and brittleness of solids. According to Pugh’s criterion (Poisson’s criterion), if a material shows B/G > 1.75 (*ν* > 0.26), it means that this solid is ductile [51,55]. On the contrary, the solid is brittle. Table 1 shows that, at equilibrium states, CsNa_2_Bi and CsK_2_Bi are brittle (in VRH approximation). It is noteworthy that under hydrostatic compression (V/V_0_ = 0.97) and tension (V/V_0_ = 1.03), these compounds remain in the brittle regime. The degree of directionality of the covalent bonds can be estimated from the value of Poisson’s ratio. The value of Poisson’s ratio is small (*ν* = 0.1) for *covalent* materials, while for *ionic* materials, a typical value of *ν* is 0.25. Poisson’s ratio values of CsNa_2_Bi and CsK_2_Bi are about *ν* < 0.18 and *ν* > 0.23, respectively. Therefore, the bonds in CsNa_2_Bi and CsK_2_Bi compounds are dominated by the covalent and ionic contributions, respectively, and the covalent contribution increases with hydrostatic tension.

**Figure 3:**
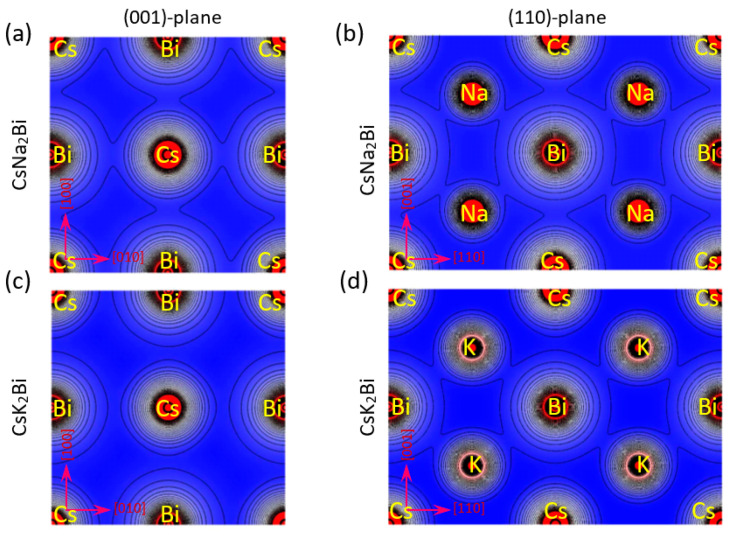
Electronic charge densities in (100) and (110) planes for (**a**,**b**) CsNa_2_Bi and (**c**,**d**) CsK_2_Bi compounds. It is evident from the valence charge density contour of the CsNa_2_Bi compound that the Bi charge density overlaps with Na and Cs, pointing to a covalent bond. In addition, the Cs-atom of CsK_2_Bi compound in the (001)-plane has a spherical electron charge density distribution with overlap with Bi and K, pointing to an ionic bond.

To explain the nature of chemical bonding in the different atoms, the valence electronic charge density distribution was computed in (100) and (110) crystallographic planes at equilibrium states of the CsNa_2_Bi and CsK_2_Bi compounds (see Figure 3). It is evident from the valence charge density contours of Figure 3a,b that the Bi charge density overlaps Na and Cs alkali metals in the CsNa_2_Bi compound, pointing to a covalent bond. In addition, it can be seen from Figure 3c that Cs in the CsK_2_Bi compound, in the (001) and (110) planes, have a spherical electron charge density distribution with no overlap with the Bi and K atoms, pointing to an ionic bond. However, in this compound, some overlap exists between K and Bi atoms in the (110)-plane, which points to a covalent bond (Figure 3d). Thus, the covalent nature of the atomic bonds in the CsNa_2_Bi compound is more pronounced than in the CsK_2_Bi compound. On the other hand, the presence of s-p hybridization between alkali metals and bismuth could be further confirmation of the presence of a covalent bond between these atoms, which can also be seen in the electron density map. These results are consistent with Poisson’s ratio analysis.

Another important mechanical parameter is the Kleinman parameter (*ξ*), which describes the relative positions of the cation and anion sublattices under volume-conserving strain distortions for which positions are not fixed by symmetry [56]. The internal strain can be quantified by *ξ*. This parameter describes the relative ease of bond bending versus bond stretching. In general, minimizing bond bending leads to *ξ* = 0, while minimizing bond stretching leads to *ξ* = 1. The Kleinman parameter is defined as the elastic constants by the following equation:

(6) $$\xi =\frac{C_{11}+8C_{12}}{7C_{11}+2C_{12}}.$$

The Kleinman parameter under V/V_0_ = 1.03, V/V_0_ = 1.0, and V/V_0_ = 0.97 are found to be 0.446, 0.454, and 0.392 (1.111, 0.930, and 0.665) for CsNa_2_Bi (CsK_2_Bi), respectively. Therefore, the value of *ξ* for CsNa_2_Bi (CsK_2_Bi) indicates that bond bending (bond stretching) is dominated in this compound.

**Figure 4:**
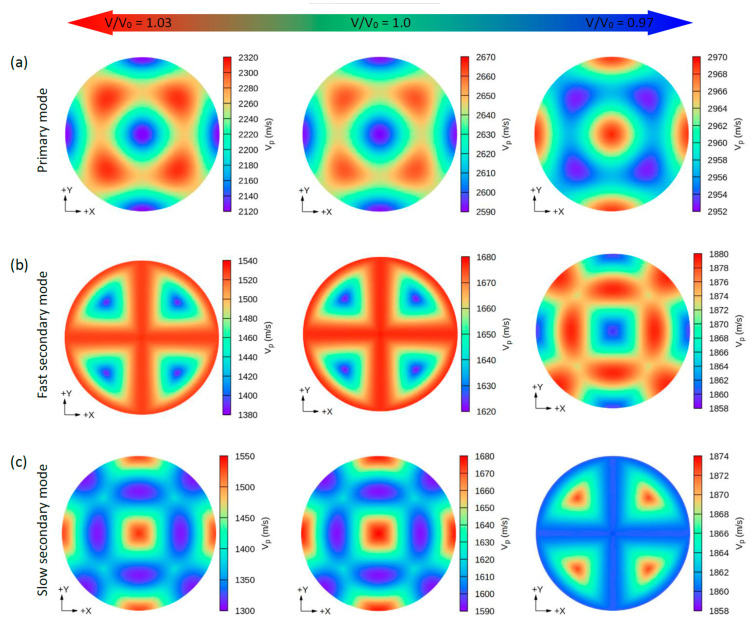
The calculated 2D directional dependence of the phase wave velocity (V_p_) in (xy)/(001)-plane for CsNa_2_Bi. There are two types of acoustic wave velocities, i.e., the longitudinal wave velocity and the transverse wave velocities in two directions. The single primary mode (**a**) is the longitudinal wave velocity, and the two secondary modes, namely, fast secondary (**b**) and slow secondary (**c**), correspond to the transverse wave velocities. Although under hydrostatic tension no changes are observed in the propagation patterns of V_p_, in hydrostatic compression, these patterns change significantly.

**Figure 5:**
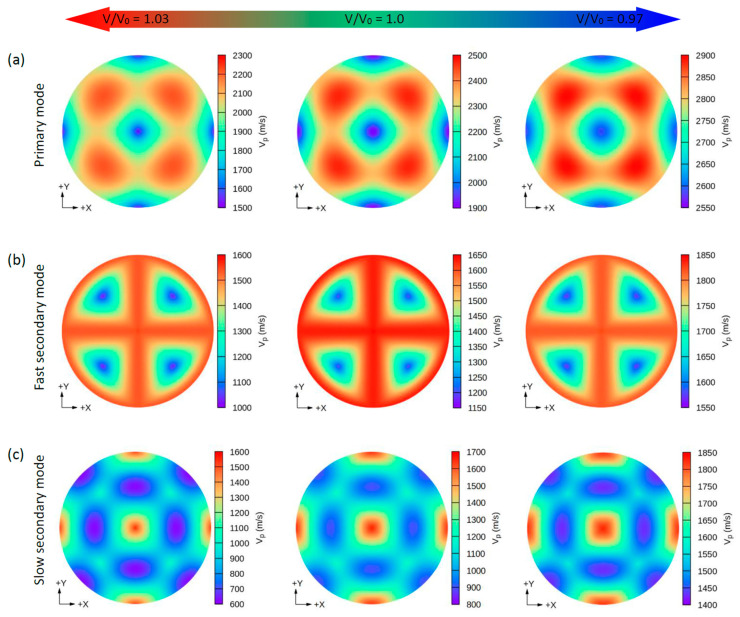
The calculated 2D directional dependence of the phase wave velocity (V_p_) in (xy)/(001)-plane for CsK_2_Bi. There are two types of acoustic wave velocities, i.e., the longitudinal wave velocity and the transverse wave velocities in two directions. The single primary mode (**a**) is the longitudinal wave velocity and the two secondary modes, namely, fast secondary (**b**) and slow secondary (**c**), correspond to the transverse wave velocities.

**Figure 6:**
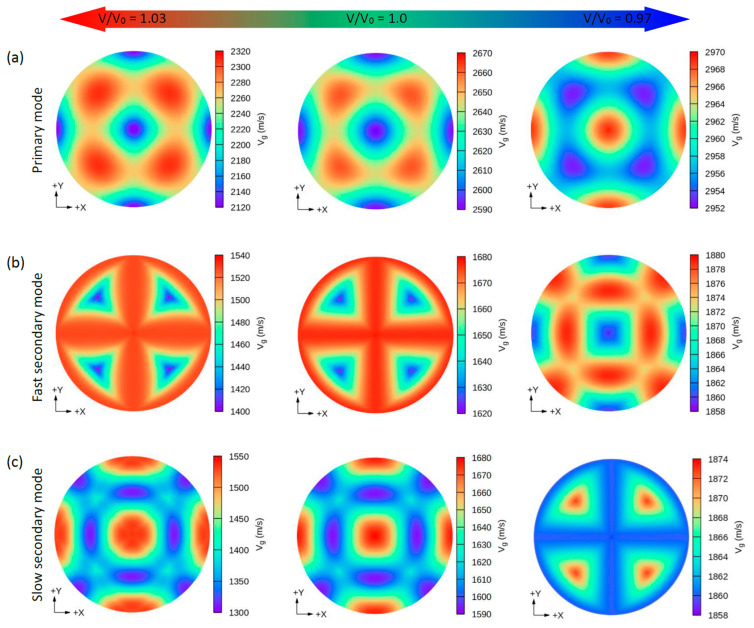
The calculated 2D directional dependence of the group wave velocity (V_g_) in (xy)/(001)-plane for the CsNa_2_Bi compound. There are two types of acoustic wave velocities, i.e., the longitudinal wave velocity and the two transverse wave velocities in two directions. The single primary mode (**a**) is the longitudinal wave velocity and the two secondary modes, namely, fast secondary (**b**) and slow secondary (**c**), correspond to the transverse wave velocities. Although under hydrostatic tension no change is observed in the propagation patterns of the V_g_, in hydrostatic compression, these patterns change significantly.

**Figure 7:**
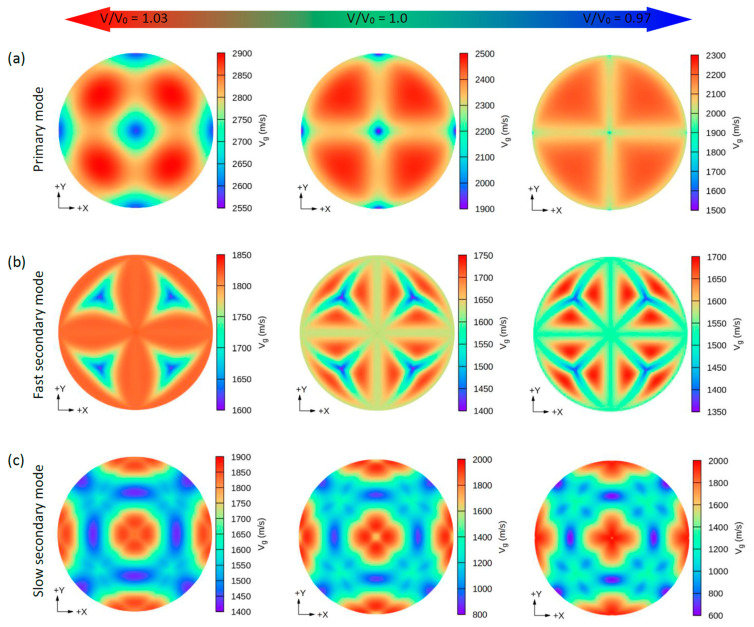
The calculated 2D directional dependence of the group wave velocity (V_g_) in (xy)/(001-plane for CsK_2_Bi. There are two types of acoustic wave velocities, i.e., the longitudinal wave velocity and the transverse wave velocities in two directions. The single primary mode (**a**) is the longitudinal wave velocity and the two secondary modes, namely, fast secondary (**b**) and slow secondary (**c**), correspond to the transverse wave velocities.

Due to the small values of the elastic constants of these structures, other properties such as the group wave velocities (V*_g_*) and the phase wave velocities (V*_p_*) may be interesting. Using the elastic constants and the density of these compounds, we can further determine the direction dependence of these properties. We calculated the V*_g_* and V*_p_* from elastic constants using the *Christoffel* equation [57] for both the longitudinal (L) wave velocity and the two transverse (T) modes. The two secondary modes, namely, the fast secondary mode (FS) and slow secondary mode (SS), correspond to the T-wave, and the single primary mode (P) is the L-wave [57]. Comparing these properties provides a measure of how the acoustic properties deviate from isotropy and allows for a direct comparison of the anisotropy among different materials. Figure 4, Figure 5, Figure 6 and Figure 7 show the calculated directional-dependent group and phase velocities of Cs(Na, K)_2_Bi for the primary and secondary modes at different pressures. It is observed that both compounds in the equilibrium state have approximately similar patterns in primary modes of phase velocity (Figure 4a and Figure 5a). This pattern has not changed in either compound after applying hydrostatic tension (V/V_0_ = 1.03), and only the maximum and minimum values of the P mode have been reduced. Under V/V_0_ = 1.0 and V/V_0_ = 1.03, V*_p_* has maximum (minimum) values in the P mode along the [111] ([100], [010], and [001]) direction(s). In addition, V*_p_* has maximum (minimum) values in the FS and SS modes along the (110)/(011)/(101) plane ([111] direction) and [100]/[010]/[001] direction ([110]/[101]/[011] direction), respectively (Figure 4b,c). For the two secondary modes (i.e., FS and SS modes) in an equilibrium state and under hydrostatic tension, the patterns have not changed, and the minimum and maximum values are in the [111] and [100] ([001] or [010]) directions, respectively. Although under hydrostatic tension, the change is not observed in the propagation patterns of the V*_p_*, under hydrostatic compression, these patterns change significantly. For the CsNa_2_Bi under hydrostatic compression (V/V_0_ = 0.97), the propagation patterns of P, FS, and SS modes of V*_p_* are reversed so that the direction of the minimum and maximum values are switched, as shown in Figure 4. As shown in Figure 5, such behavior does not exist in the phase velocity propagation pattern of CsK_2_Bi.

Similar to the phase velocity, the group velocity behavior is shown in Figure 6 and Figure 7. However, a few points are worth mentioning. Under hydrostatic compression of CsK_2_Bi, the maximum value for the P mode of V*_g_* in the [001] (or [010] and [100]) direction is sharpened, while in the equilibrium state (or hydrostatic tension) in the [001] direction, it covers a large area (see Figure 6a compared to Figure 7a). Under this pressure (V/V_0_ = 1.03) for the FS mode of V*_g_*, the distribution pattern is much more complex in CsK_2_Bi than in the case of the FS mode at hydrostatic tension (V/V_0_ = 0.97) (see Figure 6a compared to Figure 7b). According to these results, it can be seen that the group and phase velocities of CsNa_2_Bi are sensitive to hydrostatic compression, while those of CsK_2_Bi are not. The minimum and maximum values of phase and group velocities for the three propagation modes (P, FS, and SS) decrease and increase under hydrostatic tension and hydrostatic compression, respectively. These values are listed in Table 2.

### 3.3. Elastic Anisotropy

For practical applications of solid materials, like mechanical properties, knowledge about the anisotropic nature of these elastic properties is vital. As mentioned in the previous sections, the elastic anisotropy of materials is responsible for certain essential physical phenomena, such as crack behavior, phase transformations, anisotropic plastic deformation, etc. The extent of anisotropy can be determined from the different values of elastic parameters in different crystallographic directions and anisotropy indices. Therefore, in the continuation of this section, we will focus on the anisotropic elastic properties of Cs(Na, K)_2_Bi and the effect of hydrostatic pressure on them. For this purpose, the illustrations of Young’s modulus and Poisson’s ratio in the different crystal planes and three-dimensional closed surfaces are computed using the El*A*Tools code. The behavior of anisotropy is understood from the shape of the three-dimensional (3D) plots. For isotropic materials, the 3D diagrams of these elastic parameters are expected to be perfectly spherical and their projections on different planes to be circular. Thus, the deviation from spherical and circular shapes represents the anisotropic nature. In addition to this method, some anisotropy indices are explored due to their scientific interest. The universal anisotropy index (*A*^U^) and the Zener anisotropy factor (*A*^Z^) are the most critical anisotropy indices to describe elastic anisotropy. The following equations (Equations (7) and (8)) were used to calculate these anisotropic indexes, and the outcomes are listed in Table 2.

(7) $$A^{U}=5\frac{G_{V}}{G_{R}}+\frac{B_{V}}{B_{R}}-6\;,$$

(8) $$A^{Z}=\frac{2C_{44}}{C_{11}-C_{12}}.$$

**Figure 8:**
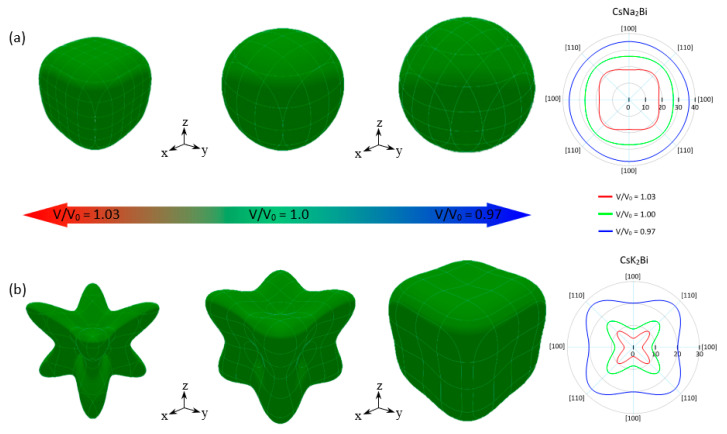
3D representation and 2D projection (in xy-plane) of Young’s modulus of CsNa_2_Bi (**a**) and CsK_2_Bi (**b**) in equilibrium state (V/V_0_ = 1.0), under hydrostatic compression (V/V_0_ = 0.97), and tension (V/V_0_ = 1.03). The behavior of anisotropy is understood from the shape of the three-dimensional (3D) plots. For isotropic materials, the 3D diagrams of Young’s modulus are expected to be perfectly spherical and its projections on different planes to be circular. The deviation from the spherical and circular shapes indicates anisotropy.

If a solid presents *A*^U^ = 0 and *A*^Z^ = 1, the solid exhibits an isotropic nature; otherwise, the solid is anisotropic. In addition, the larger values of *A*^U^ and *A*^Z^ indicate a higher degree of elastic anisotropy. The orientation dependence and two-dimensional (2D) representation in the *xy*-plane of Young’s modulus of Cs(Na, K)_2_Bi under V/V_0_ = 1.03, 1.0, and 0.97 are plotted in Figure 8. As shown in Figure 8a, CsNa_2_Bi in the equilibrium state exhibits a relatively isotropic nature with *A*^U^ = 0.0126 and *A*^Z^ = 1.11. Under hydrostatic compression (V/V_0_ = 0.97), this compound is completely isotropic. This is because its planar contours are more spherical than the equilibrium state. Anisotropy indices *A*^U^ ≈ 0 and *A*^Z^ = 0.98 also confirm this. Hydrostatic compression increases the anisotropy of this compound and takes the anisotropy indices out of the isotropic criteria. The degree of anisotropy of CsK_2_Bi is higher than CsNa_2_Bi, as shown in Figure 8b. This is due to the difference between the minimum (E*_min_*) and maximum (E*_max_*) values of Young’s modulus (E) (see Table 2), and the 3D graphs (2D projections) close to the sphere (circular). It should be noted that the E*_max_* (E*_min_*) is on in (110) ((100)) and [111] ([100]) directions. These results have also been proven by the elastic anisotropy indices *A*^U^ and *A*^Z^ in Table 2. Similar to CsNa_2_Bi, hydrostatic compression (tension) reduces (increases) the degree of anisotropy.

**Figure 9:**
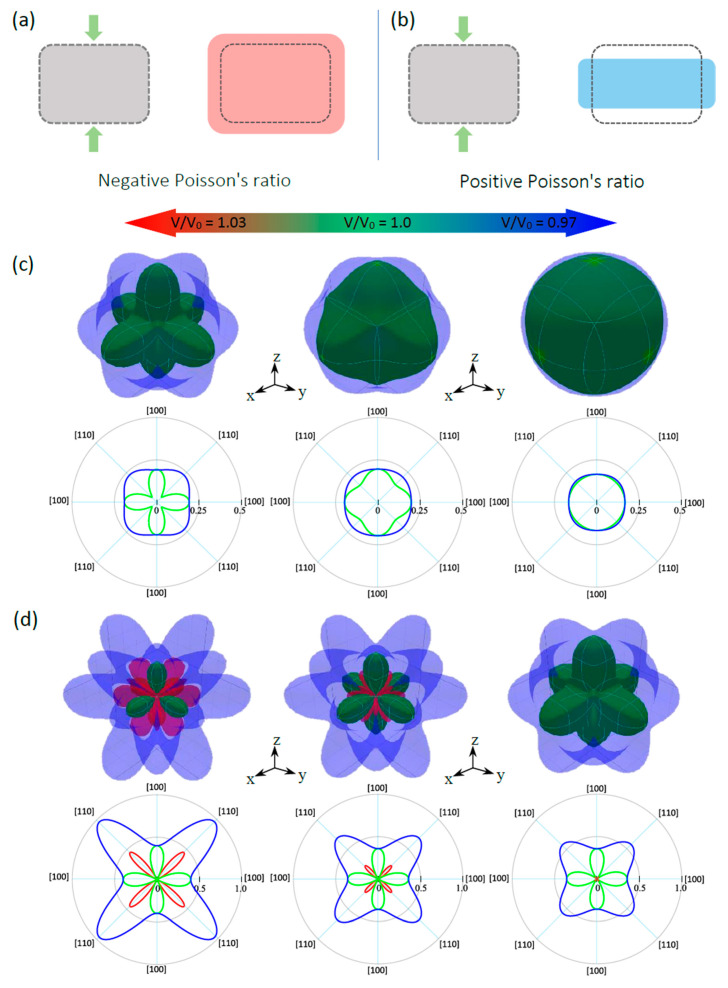
Schematic representation of (**a**) positive and (**b**) negative Poisson’s ratios of materials. Green arrows represent the direction of the tension. 3D representation and 2D projection (in xy-plane) of calculated Poisson’s ratios of (**c**) CsNa_2_Bi and (**d**) CsK_2_Bi. Green (red) color corresponds to the positive (negative) values of Poisson’s ratio.

Materials that have NPR are known as auxetic materials. These materials have attracted special attention due to their exceptional advantages in sensing technologies. Poisson’s ratio is the ratio of the transverse contraction strain to the longitudinal extension strain in the direction of the stretching force (see Figure 9a). Therefore, a material with NPR expands in the transverse direction (TD) when stretched in the longitudinal direction (LD) (see Figure 9b). Interestingly, in addition to the anisotropic nature of CsNa_2_Bi and CsK_2_Bi, CsK_2_Bi exhibits an auxetic property, although this property has only been observed in cubic elemental metals so far [58]. Using the elastic constants, we have analyzed the spatial variation of Poisson’s ratio for each of the studied compounds. In this analysis, the spherical coordinates of Poisson’s ratio, *ν*(*θ*; *φ*; *χ*), require an extra dimension in addition to the *θ*(0; *π*) and *φ*(0; 2*π*) coordinates. This additional dimension can be characterized by the angle *χ*(0; 2*π*).

The results in the equilibrium state and under hydrostatic pressure are shown in Figure 9c,d. The blue color in these figures represents the (001) surface obtained at the maximum of χ(0; 2π), while the red and green lines correspond to the negative and positive values of *ν* obtained at the minimum of *χ*, respectively. As can be seen, in the equilibrium state (V/V_0_ =1.0), CsNa_2_Bi and CsK_2_Bi have positive and negative Poisson’s ratios, respectively. It is noteworthy that CsNa_2_Bi is not an auxetic material, and as stated, this compound is almost isotropic. At the equilibrium state, when the TD is parallel to the [110] direction in CsNa_2_Bi, the maximum (minimum) positive Poisson’s ratio is close to 0.16 (0.11). In CsK_2_Bi, when the TD is parallel to the [100] (or [010]) direction, a maximum or minimum positive Poisson’s ratio of 0.36 (see 2D projection in *xy*-plane in Figure 9d) can be reached (blue and green colors), while the maximum negative Poisson’s ratio (red color) is −0.22 when the TD is along the [111] direction (45). Hydrostatic pressures have attractive effects on the auxetic and anisotropic nature of these compounds. At hydrostatic tension, the anisotropy of Poisson’s ratio is illustrated in Figure 9c, where the positive maximum (0.23) and minimum (0.04) values are observed along [110] for CsNa_2_Bi. Considering the 3D representation and 2D projection of Poisson’s ratio and the increase in the difference between the minimum (*ν*_min_) and maximum (*ν*_max_) values (see Table 2), it can be concluded that the degree of anisotropy of Poisson’s ratio increased. Since the *ν*_min_ is close to zero, it is predicted that increasing the hydrostatic tension (V/V_0_ > 1.03) in this compound will lead to the appearance of a negative Poisson’s ratio. Like CsNa_2_Bi, for the case of CsK_2_Bi, under hydrostatic tension, anisotropy of the Poisson’s ratio increases and exhibits a large negative Poisson’s ratio with a maximum value of −0.45 in the [111] direction (Figure 9d). In contrast to the hydrostatic tension, CsNa_2_Bi is almost isotropic (3D representation of *ν* is relatively spherical) at hydrostatic compression, so the *ν*_min_ (= 0.176) and *ν*_max_ (= 0.164) are close to each other. The isotropic Poisson’s ratio of this material shows an interesting concept: When the transverse contraction is parallel to a particular direction, the vertical response is the same in all directions. The effect of this pressure in CsK_2_Bi also causes the transition from an auxetic material to a non-auxetic material. This is because the NPR is almost zero (Figure 9d). It can be predicted that with increasing hydrostatic compression (V/V_0_ < 0.97), this value will be completely zero.

## 4. Conclusions

We investigated the stability, elastic, and anisotropic elastic properties of the so-far hypothetical Cs(Na, K)_2_Bi compounds under hydrostatic compression and tension using first-principles calculations. The stability checks meet the three critical conditions for thermodynamic, mechanical, and dynamic stability, evidencing highly stable compounds for practical applications. The hydrostatic compression and tension based on volumetric change of V/V_0_ = 1.0 ± 0.03 were used to investigate the mechanical properties and elastic wave velocities of these compounds. The results show that these compounds are brittle in an equilibrium state (V/V_0_ = 1.0) and under the studied pressures. The compounds are sensitive to the type of hydrostatic pressure, with interesting behaviors appearing in their mechanical properties. In CsNa_2_Bi, the direction (propagation pattern) of the elastic wave velocity is switched (changed) under hydrostatic compression (V/V_0_ = 0.97), whereas under hydrostatic tension (V/V_0_ = 1.03), such behavior is not observed. On the other hand, in CsK_2_Bi, there is no significant change in the propagation pattern of elastic waves, and only the minimum and maximum values change. Hydrostatic compression and tension in both compounds reduce and increase the mechanical anisotropy, respectively. The anisotropy index and the spatial shape of Young’s modulus show that CsNa_2_Bi has complete isotropic behavior under hydrostatic compression with good approximation. The results obtained for CsK_2_Bi show that it has a high anisotropic nature and is an auxetic material at equilibrium. Hydrostatic compression eliminates the NPR in this compound. These compounds offer promising candidates for the design and development of high-performance nanoscale electromechanical devices.

## Figures and Tables

**Table 1:** Calculated elastic constants (Cij), bulk modulus (B), shear modulus (G), Young’s modulus (E), and Kleinman parameter (ξ) of Cs(Na, K)_2_Bi compounds under hydrostatic compression (V/V_0_ = 0.97), tension (V/V_0_ = 1.03), and equilibrium state (V/V_0_ = 1.0). Voigt, Reuss, and Voigt–Reuss–Hill were utilized to calculate these moduli.

Properties	CsNa_2_Bi			CsK_2_Bi		
	V/V_0_ = 1.03 (~0.76 GPa)	V/V_0_ = 1.0	V/V_0_ = 0.97 (~1.0 GPa)	V/V_0_ = 1.03 (~0.72 GPa)	V/V_0_ = 1.0	V/V_0_ = 0.97 (~1.0 GPa)
**C_11_ (GPa)**	19.82	29.58	38.81	8.80	14.05, 14 *	25.88
**C_12_ (GPa)**	4.73	7.24	7.71	5.83	7.85, 6 *	10.14
**C_44_ (GPa)**	10.26	12.38	15.23	9.09	10.25, 9 *	12.73
**B_V_/B_R_/B_VRH_ (GPa)**	9.7/9.7/9.7	14.7/14.7/14.7	18.1/18.1/18.1	6.8/6.8/6.8	9.9/9.9/9.9	15.4/15.4/15.4
**G_V_/G_R_/G_VRH_ (GPa)**	9.1/8.9/9.0	11.9/11.9/11.9	38.6/38.6/38.6	6.0/2.9/4.5	7.39/5.32/6.36	10.9/10.2/10.5
**E_V_/E_R_/E_VRH_ (GPa)**	20.9/20.6/20.7	28.1/28.1/28.1	35.9/35.9/35.9	14.0/7.8/10.9	17.7/13.5/15.6	26.2/25.1/25.6
**ν_v_/ν_R_/ν_VRH_**	0.142/0.148/0.145	0.181/0.181/0.181	0.169/0.169/0.169	0.158/0.309/0.233	0.201/0.272/0.236	0.215/0.228/0.222
**B/G_V_/B/G_R_/B/G_VRH_**	1.06/1.08/1.07	1.23/1.23/1.23	1.17/1.17/1.17	1.12/2.29/1.51	1.34/1.86/1.55	1.42/1.50/1.46
***ξ***	0.446	0.454	0.392	1.111	0.930	0.665

* Taken from Materials Project with mp-867339 ID.

**Table 2:** The minimum (max) and the maximum (min) values of primary mode (P), fast secondary (FS), and slow secondary (SS) of the phase and group velocity, Young’s modulus (E), Poisson’s ratio (ν), and anisotropic indexes A^U^ and A^Z^ of Cs(Na, K)_2_Bi under hydrostatic compression (V/V_0_ = 0.97), hydrostatic tension (V/V_0_ = 1.03), and equilibrium state (V/V_0_ = 1.0).

Proprieties	CsNa_2_Bi			CsK_2_Bi		
	V/V_0_ = 1.03	V/V_0_ = 1.0	V/V_0_ = 0.97	V/V_0_ = 1.03	V/V_0_ = 1.0	V/V_0_ = 0.97
$P_{max}^{p}\;$ **(m/s)**	2307.4	2661.1	2968.6	2213.9	2470.7	2894.1
$P_{min}^{p}\;$ **(m/s)**	2121.3	2591.6	2952.7	1533.5	1906.5	2587.8
$FS_{max}^{p}\;$ **(m/s)**	1526.6	1676.3	1878.8	1533.5	1628.8	1815.2
$FS_{min}^{p}\;$ **(m/s)**	1386.0	1621.3	1859.9	1023.4	1194.0	1568.6
$SS_{max}^{p}\;$ **(m/s)**	1526.6	1676.3	1872.5	1509.2	1628.8	1815.2
$SS_{min}^{p}\;$ **(m/s)**	1308.7	1592.6	1859.9	619.4	895.0	1426.8
$P_{max}^{g}\;$ **(m/s)**	2307.4	2661.1	2968.6	2213.9	2470.7	2894.1
$P_{min}^{g}\;$ **(m/s)**	2121.3	2591.6	2952.7	1533.5	1906.5	2587.8
$FS_{max}^{g}\;$ **(m/s)**	1526.6	1676.3	1878.8	1686.4	1717.5	1818.4
$FS_{min}^{g}\;$ **(m/s)**	1401.9	1623.2	1859.9	1368.8	1401.2	1613.8
$SS_{max}^{g}\;$ **(m/s)**	1530.4	1676.3	1872.6	1949.9	1932.2	1853.8
$SS_{min}^{g}\;$ **(m/s)**	1308.7	1592.6	1859.9	619.4	895.0	1426.8
**E_max_ (GPa)**	22.80	28.99	36.26	29.95	22.87	18.87
**E_min_ (GPa)**	18.00	26.74	35.69	20.17	8.41	4.15
***ν_max_***	0.230	0.209	0.176	0.373	0.682	0.961
***ν*** ***_min_***	0.040	0.147	0.164	0.049	−0.220	−0.449
**A^U^**	0.1148	0.0126	0.0005	5.1503	1.9368	0.2837
**A^Z^**	1.36	1.11	0.98	6.12	3.30	1.61

## Data Availability

The data presented in this study are available upon request from the corresponding author.
